# Moving adsorption belt system for continuous bioproduct recovery utilizing composite fibrous adsorbents

**DOI:** 10.3389/fbioe.2023.1135447

**Published:** 2023-06-01

**Authors:** Yijia Guo, Martin Kangwa, Wael Ali, Thomas Mayer-Gall, Jochen S. Gutmann, Claus Zenneck, Martina Winter, Alois Jungbauer, Hector Marcelo Fernandez Lahore

**Affiliations:** ^1^ School of Science, Jacobs University Bremen gGmbH, Bremen, Germany; ^2^ Deutschen Textilforschungszentrum Nord-West gGmbH, Krefeld, Germany; ^3^ Department of Physical Chemistry, Center for Nanointegration (CENIDE), University of Duisburg-Essen, Essen, Germany; ^4^ MDX Biotechnik International GmbH, Nörten-Hardenberg, Germany; ^5^ Department of Biotechnology, Institute of Bioprocess Science and Engineering, University of Natural Resources and Life Sciences, Vienna, Austria; ^6^ Unit Biotechnologies, Department of Environmental Research and Innovation, Luxembourg Institute of Science and Technology, Esch-sur-Alzette, Luxembourg

**Keywords:** nylon 6, strong cation-exchanger, moving belt system, continuous bioproduct recovery, Humira monoclonal antibody

## Abstract

A continuous protein recovery and purification system based on the true moving bed concept is presented. A novel adsorbent material, in the form of an elastic and robust woven fabric, served as a moving belt following the general designs observed in known belt conveyors. The composite fibrous material that forms the said woven fabric showed high protein binding capacity, reaching a static binding capacity equal to 107.3 mg/g, as determined via isotherm experiments. Moreover, testing the same cation exchange fibrous material in a packed bed format resulted in excellent dynamic binding capacity values (54.5 mg/g) even when operating at high flow rates (480 cm/h). In a subsequent step, a benchtop prototype was designed, constructed, and tested. Results indicated that the moving belt system could recover a model protein (hen egg white lysozyme) with a productivity up to 0.5 mg/cm^2^/h. Likewise, a monoclonal antibody was directly recovered from unclarified CHO_K1 cell line culture with high purity, as judged by SDS-PAGE, high purification factor (5.8), and in a single step, confirming the suitability and selectivity of the purification procedure.

## 1 Introduction

Resin-based chromatography is a common unit operation often employed in the downstream processing of biopharmaceuticals, usually accounting for over 50% of the total manufacturing costs (Hardick et al., 2013; Hardick et al., 2015). Packed-bed chromatography requires an extensively clarified feed stream so as to prevent clogging and consequent high-pressure drops across the column. Moreover, with beaded adsorbents, a rather slow intra-particle diffusion process can hinder productivity due to limited operational flow rates (Guo et al., 2022). By replacing resin-based adsorbents with fabric/fiber-based adsorbent materials, the mentioned limitations could be avoided. This is due to the mechanical stability and the open structure of fibrous materials, where a convective flow dominates even under high operational flow rates (Gholampour and Ozbakkaloglu, 2020).

Relying on a wide range of production methods and raw materials, fibrous materials are often discussed as promising and cost-effective backbone materials, with available options for surface modification to gain functional properties (Naeimirad et al., 2018). For example, Gavara et al. (2012) proposed the utilization of composite adsorbent fibers for protein chromatography. Well-designed functional group conjugation onto fibers resulted in disposable devices for immunoglobulin purification (Carbajal et al., 2020). Additionally, fibrous materials are known for their high voidage, low pressure drops (Zhang et al., 2019), biocompatibility (Ying et al., 2007), and scale-up potential (Ghosh, 2002).

The introduction of sulfonic groups onto grafted polysulphone hollow-fibers was assessed by Ventura et al. (2008); this material showed 140 g/L static binding capacity (SBC) for hen egg white lysozyme (HEWL), a swift processing time (10–15 min), and high productivity (150 g/L/h) with no size-exclusion effects present. A sulphopropyl-functionalized fibrous adsorbent based on winged shaped short-cut Nano channels™ polyethylene terephthalate filament from Allasso Industries (Raleigh, NC, USA), with more than ten-fold higher surface area over a standard round filament, was studied by Schwellenbach et al. (2016). A high SBC (90 g/L) and dynamic binding capacity (DBC, 50 g/L) for HEWL were found nearly independent of the bed-residence time, revealing negligible mass transfer limitations.

Hardick and colleagues (2015) combined diethylaminoethyl-functionalized cellulosic nanofibers with a simulated moving bed (SMB). The SMB system exploited the advantageous convective mass transfer properties of nanofiber adsorbents, providing a productivity of 3.92 g/mL/h that is considerably higher than what is normally observed with conventional beaded media. These observations opened the way for the utilization of fibrous adsorbents in continuous protein capture. However, SMB requires sophisticated hardware and complex process control strategies (Zobel-Roos et al., 2018).

True moving bed (TMB) systems are existent in the context of traditional chemical engineering operations but are scarcely utilized in process biotechnology. Muendel and Selke (1955) introduced an endless moving phosphorylated cotton belt as an adsorbent to reclaim Cu^2+^ from a diluted aqueous solution. In such a system, the mechanically driven belt cycled between loading, washing, desorption, and regeneration compartments in a shallow tank containing baffles resulting in an observed SBC equal to 3.4 mEq/g at a belt rate of 2.5 cm/min.

Hughes and Charm (1979) utilized the moving belt approach for biologicals. This system worked based on an apparatus with four contacting chambers and an affinity Mylar belt (immobilized rabbit antiserum) to purify human placental alkaline phosphatase. Similarly, Niven and Scurlock (1993) employed a soybean trypsin inhibitor immobilized nylon 66 belt to purify trypsin in a four-chamber tank with inserts in each of the chambers. An apparent trypsin transfer capacity of approx. 1–2 U/m^2^ was obtained with the specific activity of the final product being 10–40 U/mg. The mentioned experimental work demonstrated the feasibility of moving belt adsorption systems for the recovery of biologicals but also signaled several design constraints and the importance of introducing novel fibrous materials to that end.

Motived by the need to explore alternative procedures for the continuous recovery of proteins and other biological products, this work integrated a novel woven fabric adsorbent with a moving belt prototype. The system was operated in semi-continuous and fully continuous modes and tested with model proteins. Thereafter, the prototype was challenged with a whole Chinese hamster ovary K1 (CHO_K1) cell line culture producing Humira monoclonal antibody (mAb) to observe the feasibility of direct mAb recovery in the presence of cells.

## 2 Materials and methods

### 2.1 Materials

Hen egg white lysozyme (HEWL) was purchased from Afilact^®^ (Geinberg, Germany). All other chemicals were purchased in the analysis quality from AppliChem (Darmstadt, Germany), Carl Roth (Karlsruhe, Germany), Sigma-Aldrich (United Kingdom), or Honeywell Fluka™ chemicals (München, Germany) and used without further purification if not specified. All buffer solutions were filtered using a 0.45 µm cellulose acetate filter (Sartorius, Göttingen, Germany) and those for chromatographic applications were further degassed in an ultrasonic chamber (5510E-DTH, Bransonic, Missouri, United States).

Humira was produced in a recombinant monoclonal Chinese hamster ovary K1 (CHO_K1) cell line using Geneticin G-418 Sulphate (Gibco, United Kingdom) as the selection system. Fed-batch fermentations with chemically defined ActiPro™ medium (HyClone™, Austria) supplemented to 8 mM L-Glutamine were realized in DASGIP^®^ Parallel Bioreactor System (Eppendorf, Germany). Fermentation parameters were set to 60 rpm stirrer speed, 37°C, 60% dissolved oxygen, and 3 s L/h gas flow rate, and pH 7.2 was controlled by CO_2_ and 7.5% sodium bicarbonate addition. A glucose concentration of at least 2 g/L was maintained by a 10% glucose feed. Harvesting of the fermentation broth was performed on day 7 with maximum cell densities of 11 × 10^6^ cells/mL.

### 2.2 Adsorbent belt

The adsorbent belt was produced according to proprietary procedures based on a woven structure created out of nylon/dextran composite microfibers. The backbone fabric presented a surface area of ∼0.23 m^2^/g, an average fiber diameter of 15 μm, an elongation of 55.9%, and denier per filament of 1.4. The modified woven fabric possessed ion-exchange properties (Guo and Fernández-Lahore, 2022).

The measurements of static and dynamic (protein) binding capacity values were performed according to established procedures in our laboratory (Guo et al., 2022) using HEWL in phosphate buffer (PB). The methods employed to determine the degree of swelling, porosity, and zeta potential values were adopted from the same work.

A scanning electron microscope (SEM, S-3400 N II, Hitachi High-Technologies Europe GmbH, Germany) was used to examine the morphology of fibers and fabrics at an accelerating voltage of 10 kV. The surface of the fibrous materials was sputtered with gold in a vacuum for 4 min using a Quorum Emitech K500X sputter coater.

The surface area of the fabrics was measured by a nitrogen adsorption-desorption-based Brunauer–Emmett–Teller (BET) using a Gemini 2360 analyzer.

### 2.3 Belt conveyor prototype

A four-chamber belt conveyor prototype was constructed for laboratory use utilizing computer-aided design. The conveyor body was constructed by assembling discrete pieces made of acrylic glass (Firstlaser GmbH, Germany). The system accommodated a flexible and robust (moving) fabric-based cation-exchanger belt that was supported by a series of roller elements. Motion was driven employing a modified peristaltic pump (MDX Biotechnik International, Germany) acting on a centre gear via an external polyurethane (inert) belt (Mädler GmbH, Germany).

The infinite moving belt prototype was tested, keeping some critical operational parameters at constant values. The belt rate was set at 7.00 cm/min and the initial liquid volume of the contacting chambers remained equal (30 mL). The adsorptive capture of a model protein (HEWL, pI = 11.1) was implemented in a four-step process, mimicking a standard chromatographic procedure, e.g., adsorption, unbound washing, elution, and re-equilibrium. This was possible by utilising four independent chambers containing appropriate solutions, with each step taking place in one of the four chambers of the prototype (see Table 1). During operation, values for total HEWL concentration and conductivity from each of the described chambers were measured at 10 min intervals by a Biophotometer RS-232 C (Eppendorf, Hamburg, Germany) and a FiveGo F3 Portable Conductivity Meter equipped with a LE703 IP67 sensor (Mettler Toledo, Gießen, Germany), respectively.

**TABLE 1:** Prototype chambers, their content and function (chambers are numbered from left to right referring to Figure 3). PB = 20 mM pH 7.4 phosphate buffer (∼3.1 mS/cm).

Chamber #/Name	Function	Buffer
1/Sampling	Binding	Protein solution in PB
2/Washing	Unbound washing	PB
3/Elution	Desorption	PB plus desorption buffer
4/Re-equilibrium	Re-equilibrium	PB

### 2.4 Process analytics

The total protein quantification of Humira monoclonal antibody was evaluated with analytical protein A affinity chromatography according to Recanati et al. (2022). Estimation of the purification of the Humira monoclonal antibody was performed via reducing SDS-polyacrylamide gel electrophoresis (PAGE) following the protocols provided by Köppl et al. (2022).

## 3 Results and discussion

### 3.1 Characteristics and performance of the adsorbent

The composite adsorbent belt was created employing a nylon 6 woven fabric backbone that was subsequently hydrophilized and functionalised with sulphopropyl (SP) ion-exchange groups.

**FIGURE 1:**
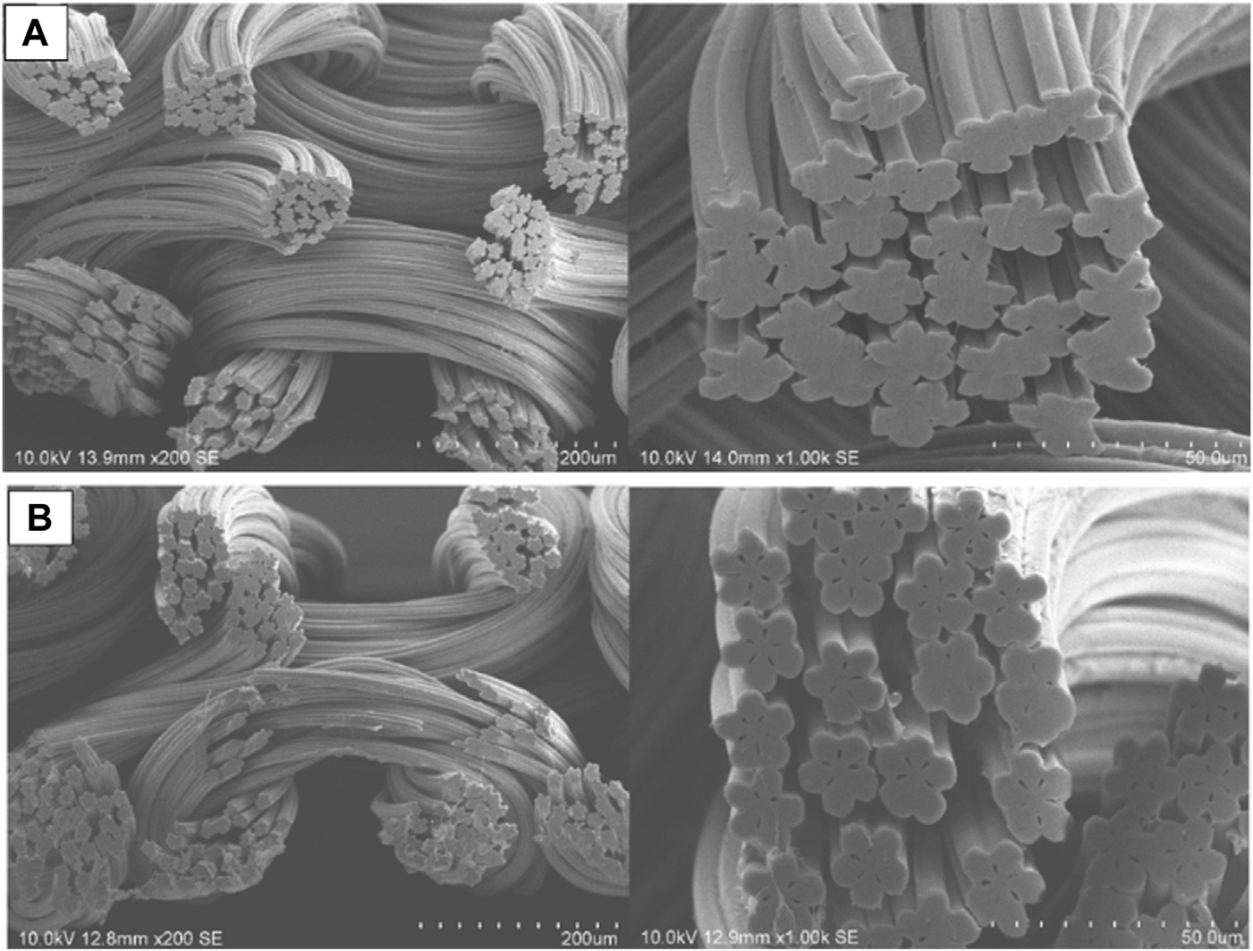
Scanning electron microscope images of **(A)** pristine (unmodified backbone) and **(B)** modified (hydrophilized and sulphopropyl functionalized backbone) nylon 6 fabrics in different scales.

Figure 1 depicts an SEM image of the pristine (unmodified) material and the resulting composite. No main structural changes were observed but a degree of roughness was introduced upon functionalisation. A five-petal cross-sectional shape was observed, a feature that provides a larger surface area in addition to other technologically relevant properties (Tascan et al., 2011; Pakravan et al., 2016; Hufenus et al., 2020). This confirmed the structural characteristics of the backbone material and its suitability to produce a functionalised composite.

**FIGURE 2:**
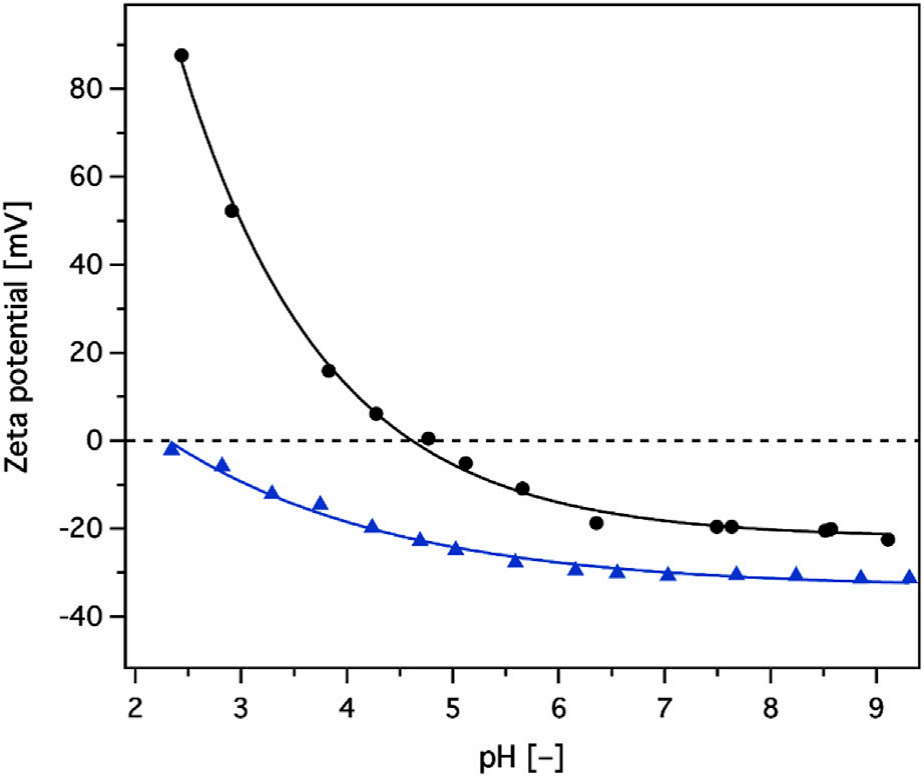
Zeta potential values, as a function of pH, for unmodified/naked backbone (black line with circles), and sulphopropyl groups introduced/modified adsorbent (blue line with triangles).

Once in contact with a liquid media of defined solution chemistry, all solid surfaces assume a zeta potential at the level of the “shear plane.” Measurements of the zeta potential values are employed to assess not only the surface charge of the solid but also its hydrophobicity (Luxbacher, 2014). Figure 2 shows the dependence of zeta potential values as a function of the pH of the contacting electrolyte solution (1 mM potassium chloride) for the nylon 6 backbone and the composite adsorbent material obtained thereof. The naked backbone had negative zeta potential values from pH 4.8 to pH 9.3, probably due to the hydrophobic nature of the nylon 6 surface attracting hydroxide ions from water that behave more hydrophobically than corresponding hydronium ions (Luxbacher, 2014). Therefore, at higher pH values, the higher concentration of hydroxide ions would produce a more negative surface charge. Functionalisation of the backbone with a hydroxyl rich layer and SP groups resulted in a clear charge profile modification with negative zeta potential values over the whole observed pH range from 2.3 to 9.3. This indicated that the composite behaves as a cation exchanger.

A charged fibrous material offering enhanced surface area could become an appropriate adsorbent material for protein capture. Therefore, the binding ability of the composite was explored using HEWL at pH 7.4. The composite material showed 4.5- and 27.3-times higher binding capacity than the backbone under equilibrium conditions (isotherm experiment, contact time = 3 h) and dynamic conditions (breakthrough curve experiment, flow rate = 60 cm/h), respectively Table 2 comparatively summarizes several relevant parameters that pertain to the backbone as opposed to the composite material. The mentioned differences in binding capacity values for the backbone and the composite could be, at least in part, attributed to a surface area increase as observed for the composite material in relation to the backbone (e.g., a 4.1-times increase). Dynamic conditions particularly favoured the potential use of the adsorbent composite since low unspecific binding on the backbone was observed (2.0 mg/g) while charge-mediated binding observed for the composite reached 54.5 mg/g. Moreover, the protein binding capacity values were almost insensitive to flow velocity. An 8-times increase in flow velocity reduced the DBC by ca. 12.9% (see Table 3). Although the composite material showed a slight decrease in DBC from 60 to 120 cm/h, which could be explained by a small increase in axial dispersion, the values were independent of flow rate after 120 cm/h until 480 cm/h, indicating convective flow. Additionally, the Peclet number of the system remained constant (>63.6), further confirming the plug flow characteristics with minimal axial mixing. The results described above indicated that the woven composite adsorbent has the required mechanical and functional characteristics to be deployed in a belt conveyor system.

**TABLE 2:** Physical properties of naked nylon 6 backbone and hydrophilized and sulphopropyl introduced nylon 6 composite adsorbent. Conditions: pH 7.4 20 mM phosphate buffer as equilibration buffer for both static and dynamic binding capacities of hen egg white lysozyme (HEWL), 2 g/L HEWL as model for the dynamic one at 60 cm/h, and 1 g/L HEWL for the static one for 3 h contact with mild shaking.

	Nylon 6 backbone	Nylon 6 composite
**Static binding capacity (**mg/g)	24.1 ± 2.2	107.3 ± 4.3
**Dynamic binding capacity (**mg/g)	2.0 ± 0.07	54.5 ± 2.0
**Degree of swelling (**g/mL)	0.84 ± 0.05	0.95 ± 0.05
**Porosity (**%)	47 ± 2	30 ± 1.3
**Specific surface area (**m^2^/g)	0.23 ± 0.01	0.95 ± 0.02

**TABLE 3:** Dynamic binding capacity (DBC) of hydrophilized and sulphopropyl introduced nylon 6 composite adsorbent at different flow rates from 60 to 480 cm/h. Conditions: pH 7.4 20 mM phosphate buffer as equilibration buffer and 2 g/L hen egg white lysozyme as model protein.

Flow rate (cm/h)	Dynamic binding capacity (mg/g)
60	54.52
119	46.58
239	45.53
477	47.49

### 3.2 Prototype design and construction

**FIGURE 3:**
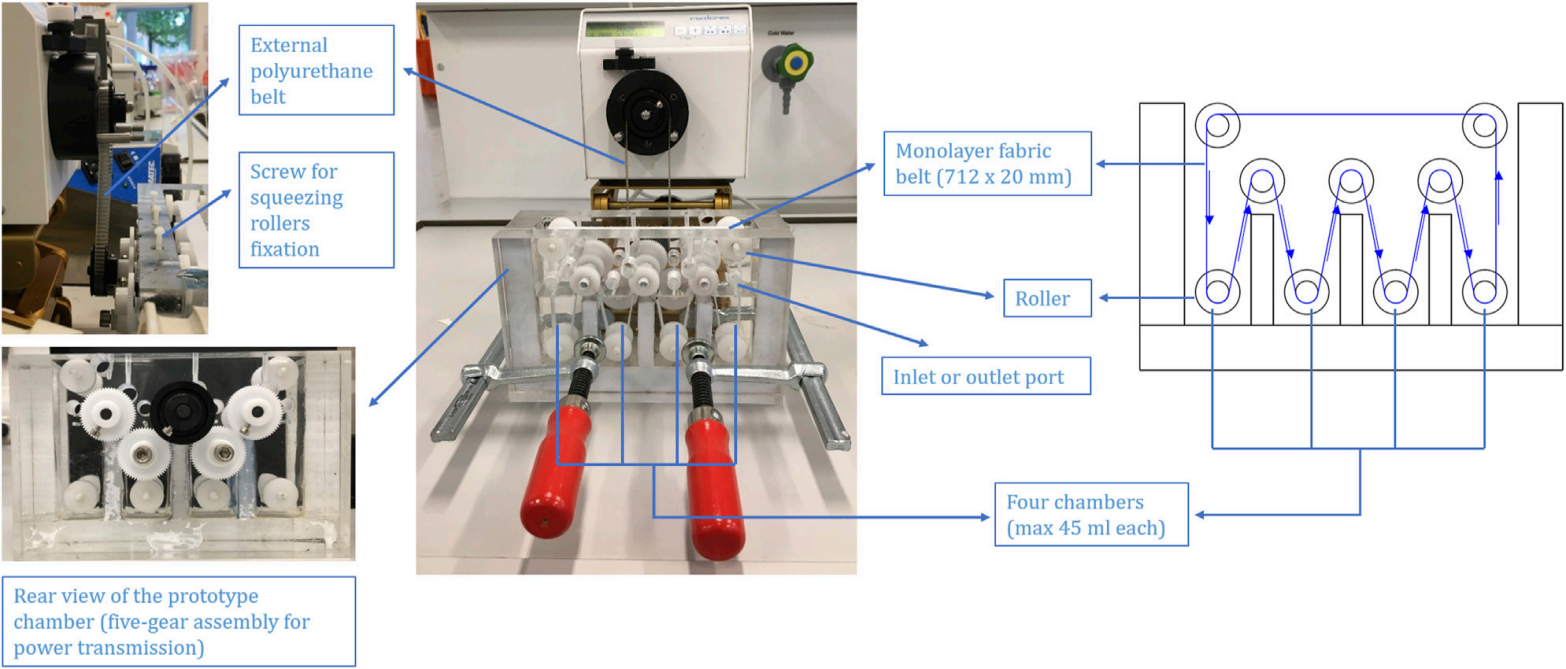
Set-up of the continuous moving belt prototype.

The concept of a continuous moving belt prototype was originally a belt conveyor consisting of a tank divided into four chambers. Each was able to possess a particular buffer volume and a particular buffer exchange speed. Because of the flexible and bendable structure of the fabric belt (monolayer, 712 × 20 mm), it passed through each chamber in sequence via rollers located at the bottom of each chamber, at the top of each chamber divider, and at the top of the tank (see Figure 3). Inlet ports for each chamber were located on the front tank wall. Outlet ports for each chamber were located on the back tank wall. Furthermore, acrylic glass was used for the construction of the tank body. For the rollers and axis of rollers, polyformaldehyde was applied for the ease of movement. A squeezing roller (not shown from Figure 3) was added adjacent to each roller in the middle row in order to squeeze the absorbed buffer out of the fabric belt back to the respective chamber. An eccentric shaft was employed here for the squeezing roller so as to adjust the gap between the squeezing roller and the respective roller to ensure that the squeezing provided was sufficient to squeeze the absorbed buffer out and simultaneously was tender enough to free the belt from too much friction created. The fixation of the squeezing rollers was accomplished by two screws for each.

A five-gear assembly outside of the tank was designed, from where three gears were introduced to the middle-row rollers which were responsible for the power transmission and a guarantee of a closed system (see Figure 3). Motions from the connected driving peristaltic pump would be transferred to the center gear (the black one in Figure 3 rear view) via an external polyurethane belt (see Figure 3). Through gear engagement, the motions were transferred to the rollers in the middle row of the prototype. Thereafter, other rollers would be driven in the direction of motion.

### 3.3 Prototype testing with model proteins (hen egg white lysozyme)

#### 3.3.1 Operation in batch and semi-continuous mode

**FIGURE 4:**
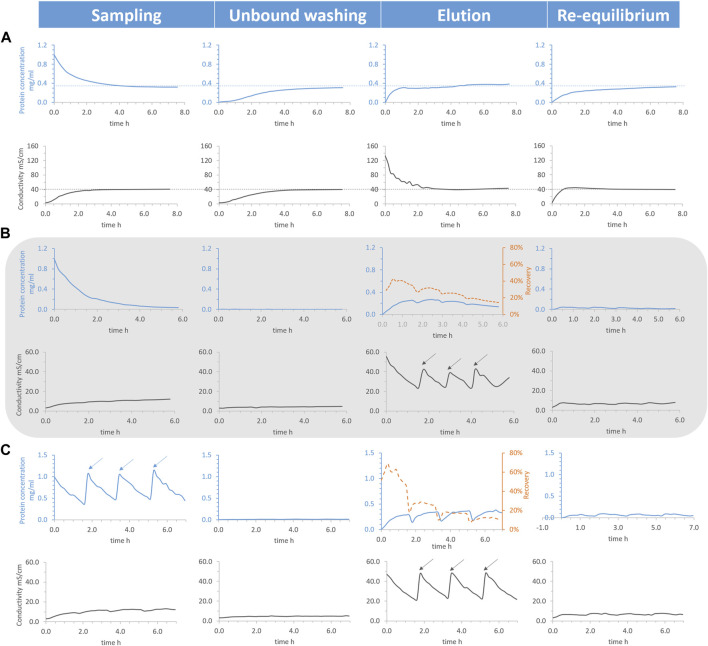
Total hen egg white lysozyme concentration (blue solid line), conductivity (black solid line), and recovery (orange dotted line) as functions of processing time. Conditions: belt rate 7.00 cm/min, pH 7.4 20 mM phosphate buffer (PB) as unbound washing and re-equilibrium buffer, 1 g/L lysozyme dissolved in the same buffer as sample, and 30 mL liquid per chamber initially. Conditions: **(A)** 2 M NaCl as disassociation buffer, **(B)** 0.75 M NaCl as disassociation buffer, and fresh PB flowing into the washing and re-equilibrium chambers continuously with a speed of 2.05 mL/min. Then, 5 mL 2 M NaCl was added into the elution chamber (marked by arrows) once the conductivity was smaller than the critical point (∼22.5 mS/cm), **(C)** 0.75 M NaCl as disassociation buffer and fresh PB flowing into the washing and re-equilibrium chambers continuously with a speed of 2.04 mL/min. A total of 15 mL was taken out from the elution chamber, 5 mL 5 g/L lysozyme was added to the sampling chamber (marked by arrows), and 20 mL 1 M NaCl was added to the elution chamber (marked by arrows) once the conductivity was smaller than the critical point.

For batch process, the total HEWL concentration soared in the elution chamber [2 M sodium chloride (NaCl) as a desorption buffer, ∼149.4 mS/cm], as expected, at the very onset (see Figure 4A). However, in spite of lower rate of increase, the total HEWL concentration of the unbound washing and re-equilibrium chambers caught the momentum, between which the re-equilibrium chamber presented a faster growth largely because of a quickly enlarged conductivity. After around 5 h, both the total HEWL concentration and conductivity from the four chambers became uniform with a final value equal to a quarter of the input (see dotted line from Figure 4A). Similar phenomena were reported from different belt rates of 3.50 and 1.75 cm/min or chamber arrangements (data not shown). This revealed that the general logistic transportation of solutes, a basic attribute of belts conveyors (McGuire, 2009), overruled the selective transportation of HEWL (ionic separation). In addition, the surplus conductivity was the other profound cause leading to the failure of the separation.

Approaches to the semi-continuous mode optimization to overcome the shortcomings above included applying 0.75 M NaCl (∼52.7 mS/cm) as an elution buffer and continuously flowing fresh PB (pH 7.4 20 mM, ∼3.1 mS/cm) into the washing and re-equilibrium chambers. With those approaches, the total HEWL concentration from the sampling chamber dropped to almost zero, only a small quantity of HEWL was detected from the washing and re-equilibrium chamber, and, apart from elution chamber, the conductivity of the other three chambers was kept lower than 12.0 mS/cm, not being able to interfere with the ionic separation (see Figure 4B). However, the total HEWL concentration of the elution chamber started decreasing after around 1.7 h, when conductivity sunk below the critical point (∼22.5 mS/cm), where selective transportation gave way to unspecific general logistic transportation. One alternative to compensate for this was to introduce 5 mL of fresh concentrated NaCl solution (2 M) into the elution chamber, as marked by arrows from the zigzag conductivity graph in Figure 4B, to reverse the decline.

The recovery of elution was calculated (orange dotted line from Figure 4B) for the semi-continuous mode, which only increased for approx. The first half hour and unfortunately sustained a steady decline afterwards. Each time after the introduction of fresh concentrated NaCl, both the recovery and the total HEWL concentration from the elution chamber underwent a slight spike. The height of the spikes diminished gradually for both as the amount of the NaCl addition increased, demonstrating that the benefits of NaCl addition decreased gradually. Following this pattern, selective transportation would be substituted for unspecific general logistic transportation eventually. On the bright side, further optimization was feasible, such as operation in continuous mode.

#### 3.3.2 Operation in continuous mode

As already known, the semi-continuous mode operation only temporarily suppressed the unspecific general logistic transportation. Approaches to further prolong the suppression and to make the process really continuous included increasing the reaction rate of the ionic separation which depends on the concentration of reactants and products. Namely, 5 mL concentrated HEWL (5 g/L) was added to the sampling chamber and 15 mL eluted HEWL was withdrawn from the elution chamber each time before NaCl addition.

Not only were synchronizing zigzag patterns found for the total HEWL concentration and conductivity in the elution chamber, but also for the total HEWL concentration in the sampling chamber (see Figure 4C). Every time after the addition of NaCl and concentrated HEWL in the respective chambers, the value of the total HEWL concentration in the elution chamber reached a similar level as before, meaning that the system was rejuvenated.

However, the calculated recovery of elution only increased for a relative short time (∼ half hour) at the beginning and sustained a steady decline afterwards. A primary reason for this could be attributed to the lack of a regeneration chamber. Part of the binding capacities of the adsorbent belt was lost during the process and could not be restored. A sharp drop in the recovery of elution at the same time as the addition of NaCl/concentrated HEWL was because of the removal of eluted HEWL.

#### 3.3.3 Operational parameters

**FIGURE 5:**
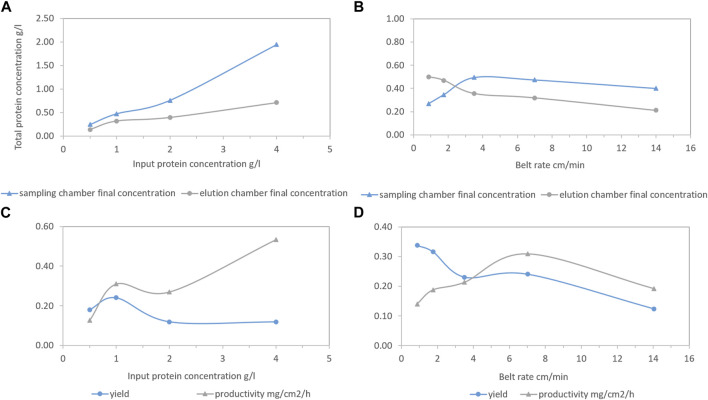
Total final hen egg white lysozyme (HEWL) concentration of the sampling chamber (blue line with triangle) and the elution chamber (grey line with circle) as functions of HEWL input concentration or belt rate **(A, B)**. Corresponding yield (blue line with circle) and productivity (grey line with triangle) as functions of HEWL input concentration or belt rate **(C, D)**. Conditions: 0.75 M NaCl as disassociation buffer, pH 7.4 20 mM phosphate buffer (PB) as unbound washing and re-equilibrium buffer, 30 mL liquid per chamber initially, and fresh PB flowing into the washing and re-equilibrium chambers continuously with speed of 2.12 mL/min. Then, 15 mL was taken out from the elution chamber, 5 mL 5 g/L HEWL was added to the sampling chamber, and 20 mL 1 M NaCl was added to the elution chamber once the conductivity was smaller than the critical point.

Different HEWL input concentrations (from 0.5 to 4.0 g/L) were tested at a fixed belt rate (7.00 cm/min). According to Figure 5A, along with the increasing HEWL input concentration, the HEWL concentration gap between the sampling and elution chambers at the end of the process became more distinct, suggesting a HEWL overload or insufficient processing time. More specifically, the process time for each protein concentration/conductivity decrease from the zigzag pattern was mainly determined by whether the conductivity of the elution chamber was above the critical point (∼22.5 mS/cm) which was determined by unspecific general logistic transportation depending on the belt rate. The higher the belt rate, the faster the protein concentration/conductivity decrease in the zigzag pattern, potentially leaving insufficient processing time (equal to protein overload). Since the belt rate was fixed, the processing time for each HEWL concentration/conductivity decrease from the zigzag pattern was fixed, leading to an adsorption limitation. Accordingly, yield decreased with increasing input protein concentration after 1 g/L, while productivity increased mainly because of the increasing total HEWL concentration in the elution chamber (see Figure 5C).

Different belt rates (0.88–14.00 cm/min) were tested at a fixed HEWL input concentration (1.0 g/L). According to Figure 5B, enough processing time determined a higher degree of the completion of ionic separation, meaning less HEWL was left in the sampling chamber and there was an increasing trend in yield (see Figure 5D). After 3.5 cm/min, the HEWL concentration gap between the sampling and elution chambers at the end of the process reached a plateau, meaning the limitation of the reaction had been reached. Moreover, a lower belt rate referred to longer processing time, generating lower productivity. The approach for a steady operation with enough time really depends on the productivity/yield required and the interaction between the protein separated and ligand utilized. As long as the kinetics of the adsorption/desorption is fast/slow enough to match the downwards course of the zigzag pattern, ensuring the majority of the target protein is fished and the time is not too slow to significantly reduce productivity, the process will work. Unfortunately, as for now, we have not found such an example, but from the purification process reported, the potential of the prototype is already obvious.

### 3.4 Purification of the Humira mAb from unclarified CHO_K1 cell line fermentation broth

The continuous moving belt prototype was challenged in continuous mode by a CHO_K1 cell line fermentation broth (13.2 mS/cm, pH 6.84) producing Humira mAb, donated by the University of Natural Resources and Life Sciences (Vienna, Austria). The purification was attempted under the following conditions: belt rate at 7.00 cm/min, 10-times diluted fermentation broth as sample, 0.75 M NaCl (∼52.7 mS/cm) as disassociation buffer, pH 5.1 20 mM phosphate buffer (∼3.5 mS/cm) as unbound washing and re-equilibrium buffer, 30 mL liquid per chamber initially, and fresh buffer flowing into the washing and re-equilibrium chambers continuously with a speed of 1.95 mL/min. In addition, 15 mL eluted Humira mAb was taken out from the elution chamber, 2.5 mL undiluted fermentation broth as sample was added to the sampling chamber, and 20 mL 1 M NaCl was added to the elution chamber, once the conductivity started out less than the critical point in the elution chamber.

**FIGURE 6:**
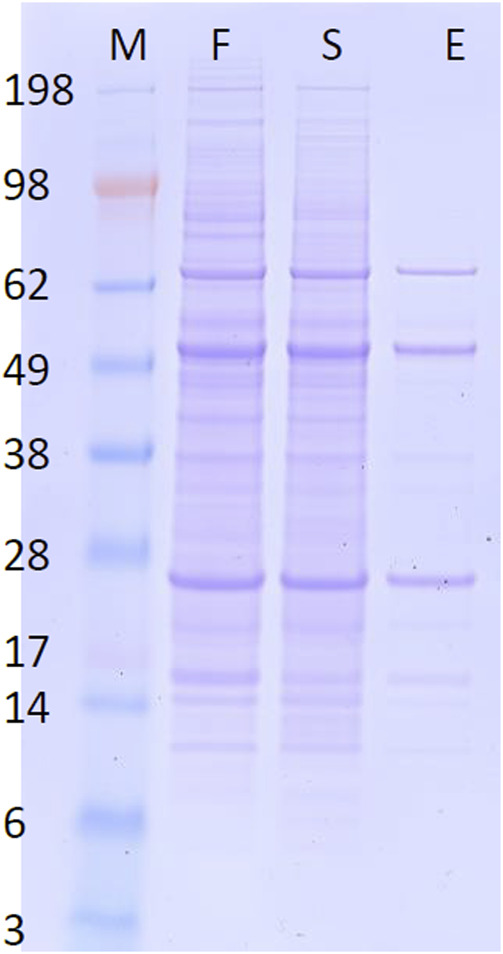
Reducing SDS-PAGE of CHO_K1 cell line fermentation broth (F), protein left in the sampling chamber at the end of the process (S), and the eluted protein from the elution chamber at the end of the process (E). M is a molecular weight marker as indicated.

Normally, mAb purification may involve several steps to attain a desired level of purity. In this example, the targeted Humira mAb was directly recovered from the fermentation broth via only one step with high purity, as ascertained by SDS-PAGE (see Figure 6). This data confirms the selectivity of the purification procedure and Humira mAb’s identity as well, since unique bands near 28 kDa (light chain) and 49 kDa (heavy chain) were clearly observed (Bandyopadhyay et al., 2015).

Various belt rates from 7.00 to 28.00 cm/min were utilized to optimize the conditions and to further understand the moving belt system. Different from utilizing HEWL as a model protein sample where selective transportation (ionic separation) and unspecific general logistic transportation (protein being transported to the following chamber physically) were indistinguishable, this case offered a new perspective where only Humira mAb can be transported via both ways, while impurities would merely be transported by the unspecific general logistic transportation. This is why the process utilizing HEWL as samples was more sensitive to the belt rate than the process utilizing Humira mAb cell culture superficially and no difference was reflected for the HEWL process applying target protein concentration or total protein concentration for the calculation of the yield and productivity (only one pillar in Table 4). Both yield and productivity would decrease at the belt rate of 28 cm/min for the HEWL process, while that was not the case for the Humira mAb cell culture process, which would climb up and then decline.

**TABLE 4:** Yield and conductivities of processes utilizing hen egg white lysozyme or Humira mAb cell culture as samples at different belt rates. For the process utilizing Humira mAb cell culture, the calculation was done by the total protein concentration and Humira mAb concentration.

Belt rate cm/min	Humira mAb cell culture				Hen egg white lysozyme	
	Total protein		Humira mAb			
	Yield (%)	Productivity mg/cm^2^/h	Yield (%)	Productivity mg/cm^2^/h	Yield (%)	Productivity mg/cm^2^/h
28.00	13.90	0.310	33.77	0.017		
14.00	14.83	0.259	22.29	0.009	12.38	0.192
7.00	10.91	0.130	63.81	0.019	24.08	0.310
3.50					23.08	0.213
1.25					31.67	0.187
0.625					33.80	0.140

When it comes to considering target protein concentrations for the calculation of the yield and productivity for two processes, no difference was reflected for the HEWL process. While, for the Humira mAb cell culture process, things were more interesting. Both yield and productivity experienced a nadir, showing different variation tendencies.

The difference between the two yields and productivities for the Humira mAb cell culture case is caused by unspecific general logistic transportation of the impurities from the cell culture. The degree of belt-fluid contact varied widely within the apparatus, which was greatly influenced by belt rate (Muendel and Selke, 1955), leading to heterogeneous suppression of unspecific general logistic transportation. An ideal contact mode between the belt and fluid would be a convective flow which is the fundamental advantage of fabric/fiber-based adsorbent over resin-based counterparts, but unfortunately was not being applied effectively here.

## 4 Concluding remarks

A mechanically-robust strong cation-adsorbent belt functionalized by SP made of woven nylon 6 fabric was fabricated with decent SBC (107.3 mg/g). The constructed fabric adsorbent possesses a specific five-petal cross-sectional shape for a given denier per filament, providing larger surface areas than round fiber. Negative zeta potential values of the constructed adsorbent belt were observed over the whole observed pH range from 2.3 to 9.3, implying the strong cationic nature. The DBC (54.5 mg/g) of the constructed adsorbent belt was independent of flow rate up to 480 cm/h, indicating convective flow with minimal axial mixing.

The developed adsorbent belts were implemented in a novel continuous purification prototype based on mechanism of belt conveyors, consisting of a four-chamber tank, wherein each chamber was responsible for association, unbound washing, elution, and re-equilibrium, respectively, and was able to possess its own buffer volume and liquid speed. Adsorbent belts passed through each chamber in sequence via rollers readily for continuous bioproduct recovery from a feedstock.

The results observed from the prototype operation revealed that the developed prototype was capable of model protein (HEWL) separation with productivity of up to 0.5 mg/cm^2^/h. Humira mAb was directly recovered from unclarified CHO_K1 cell line fermentation broth with high purity and high purification factor (5.8) via only one step, confirming the selectivity of the purification procedure.

On the other hand, certain barriers were observed during processes such as lack of a regeneration chamber and the inefficient application of convective flow. Optimization of the undesirable lack of a regeneration chamber and convective flow involves the addition of an extra chamber for adsorbent regeneration on the far right and a spray zone with fold return motion (Xiao, 2022) and spray heads. In this upgraded setup, feedstock would be sprayed onto the adsorbent belt, directly forcing fluid carrying targeted protein to go through the adsorbent belt and establishing convective flow. In order to expand the contact area, the fold return motion of the adsorbent belt would be applied. In case the spray process is too fast to finish the cationic adsorption, the distributions of spray heads are designed only for the left two-thirds of the spray zone. If this is not enough, the bottom layer of the spray zone could be employed as insurance if needed.

Not only the monolayer adsorbent application in this article but also a multiple-layer adsorbent with the same or different material and a greater surface area for belt-fluid contact is feasible if necessary. Moreover, due to the flexible structure of the belts conveyor, it is competent to pass through more rollers (more chambers) or travel farther (larger chamber volume). Scale-up is relatively easy to fulfil if the moving adsorption system is utilized for purification, which definitely deserves further attention.

## Data Availability

The original contributions presented in the study are included in the article/Supplementary Material, further inquiries can be directed to the corresponding author.
